# Antigen surface display in two novel whole genome sequenced food grade strains, *Lactiplantibacillus pentosus* KW1 and KW2

**DOI:** 10.1186/s12934-024-02296-2

**Published:** 2024-01-11

**Authors:** Kamilla Wiull, Live Heldal Hagen, Jelena Rončević, Bjørge Westereng, Preben Boysen, Vincent G. H. Eijsink, Geir Mathiesen

**Affiliations:** 1 Faculty of Chemistry, Biotechnology and Food Science, NMBU - Norwegian University of Life Sciences, Ås, Norway; 2 Faculty of Veterinary Medicine, NMBU - Norwegian University of Life Sciences, Ås, Norway

**Keywords:** *L. pentosus*, Heterologous protein expression, pSIP, Adjuvancy, Exopolysaccharides

## Abstract

**Background:**

Utilization of commensal bacteria for delivery of medicinal proteins, such as vaccine antigens, is an emerging strategy. Here, we describe two novel food-grade strains of lactic acid bacteria, *Lactiplantibacillus pentosus* KW1 and KW2, as well as newly developed tools for using this relatively unexplored but promising bacterial species for production and surface-display of heterologous proteins.

**Results:**

Whole genome sequencing was performed to investigate genomic features of both strains and to identify native proteins enabling surface display of heterologous proteins. Basic characterization of the strains revealed the optimum growth temperatures for both strains to be 35–37 °C, with peak heterologous protein production at 33 °C (KW1) and 37 °C (KW2). Negative staining revealed that only KW1 produces closely bound exopolysaccharides. Production of heterologous proteins with the inducible pSIP-expression system enabled high expression in both strains. Exposure to KW1 and KW2 skewed macrophages toward the antigen presenting state, indicating potential adjuvant properties. To develop these strains as delivery vehicles, expression of the mycobacterial H56 antigen was fused to four different strain-specific surface-anchoring sequences.

**Conclusion:**

All experiments that enabled comparison of heterologous protein production revealed KW1 to be the better recombinant protein production host. Use of the pSIP expression system enabled successful construction of *L. pentosus* strains for production and surface display of an antigen, underpinning the potential of these strains as novel delivery vehicles.

**Supplementary Information:**

The online version contains supplementary material available at 10.1186/s12934-024-02296-2.

## Background

The *Lactiplantibacillus* genus comprises numerous species that are of interest for biomedical research. The lactiplantibacilli are gram-positive, homofermentative, non-spore-forming bacteria that are nomadic residents of the human microbiome [1]. *Lactiplantibacillus* spp. are present in a variety of food products consumed by humans, such as meat, fruit and vegetables [2]. These bacteria are also used in food production and preservation and based on their nonpathogenic nature, they have the qualified presumption of safety (QPS) status [3].

*Lactiplantibacillus pentosus* (formerly known as *Lactobacillus pentosus)* is one of the most commonly isolated bacterial species from vegetables [4] and plays a crucial role in olive fermentation, due to amino acid-, short chain fatty acids-, antioxidant-, exopolysaccharide- (EPS) and vitamin production. The bacterium produces these molecules in vivo, which affects product quality and presumably adds a probiotic effect [5]. A study by Thuy et al. [6] found that three strains of *L. pentosus* exhibited tolerance when incubated at low pH or bile salts, indicating intrinsic resistance to the conditions met in the gastrointestinal tract. Various strains of *L. pentosus* have been shown to produce both tightly bound EPS (i.e. capsular polysaccharides (CPS)) and loosely associated EPS [7, 8]. Although some bacterial EPS and CPS have been related to virulence, the EPS produced by commensal lactic acid bacteria (LAB) such as *L. pentosus* have gained attention for their immunomodulating properties [8, 9]. You et al. [8] showed that EPS isolated from *L. pentosus* LZ-R-17 exhibited potent immunostimulatory activity by enhancing proliferation and cytokine production by RAW264.7 cells. This potential adjuvant effect of EPS- and CPS-producing *L. pentosus* could make strains belonging to this species attractive as novel vaccine delivery vehicles. The interest in utilization of *L. pentosus* in biomedical research has increased in recent years, with studies showing the potential of the bacterium to alleviate allergenic symptoms and DSS-induced ulcerative colon inflammation [10, 11], and a potential to attenuate *Salmonella* infections [12].

While there is increasing interest in exploiting less commonly used commensal bacteria for production and delivery of antigens [13], the potential of *L. pentosus* has hardly been explored. The importance of assessing a wide variety of bacterial strains for delivery vehicle purposes is elicited by differences in their ability to adhere to immune cells and other intrinsic immune modulating properties [14]. Bacteria from *Lactobacillaceae* such as *Lactiplantibacillus plantarum* and *Lacticaseibacillus casei* have demonstrated potential as delivery vectors in various applications, including vaccine delivery [13, 15–17]. *L. plantarum* has also been investigated for its adjuvant properties and its potential as an antigen delivery system to mucosal sites [18–20]. In contrast, *L. pentosus* has rarely been used as a delivery vehicle, despite an early study showing successful production and display of the spike protein of the transmissible gastroenteritis virus [21]. This study showed that the secreted and anchored antigen induced humoral immune responses in serum, feces and mucosal fluids in mice. These results are promising indications of the potential suitability of *L. pentosus* strains as delivery vehicles.

In the present study, we isolated two novel *L. pentosus* strains KW1 and KW2, from green olives and studied their genomic characteristics through whole genome sequencing. Furthermore, we assessed their growth rates, potential for heterologous protein production, potential adjuvant effects, and the functionality of the pSIP vector system for inducible protein production. The potential of the two strains as vaccine delivery vehicles was further evaluated by constructing recombinant KW1 and KW2 strains expressing the tuberculosis hybrid antigen Ag85B-ESAT-6-Rv2660c (H56) translationally fused to four different anchor motifs derived from the two *L. pentosus* strains.

## Results

### Genomic features of *L. pentosus* KW1 and KW2

Whole genome sequencing and assembly of the two *Lactiplantibacillus pentosus* strains resulted in circular chromosomes (KW1; 3,668,369 bp, KW2; 3,548,025 bp) in addition to four and eight circular contigs, from respectively KW1 and KW2. All except one (p8) of these circular contigs encodes one or more transposable elements and are most likely related to plasmid sequences (Table 1). The overall G + C content of the assembled chromosome is 46.4% for KW1 and 46.5% for KW2. The quality assessment of the *L. pentosus* KW1 assembly indicated a high-quality genome (99.2% complete and single-copy BUSCOs), whereas the genome assembly of *L. pentosus* KW2 encountered some fragmentation issues (91.9% complete BUSCOs, 7.3% fragmented BUSCOs) which persisted through polishing (Additional file 2: Figure S1). The fragmented BUSCOs observed in the KW2 assembly may impair gene prediction by causing frameshifts. Frameshifts in homopolymer stretches were occasionally observed during annotation of KW2.

Overall, 3539 and 3565 genes were identified in the assembled chromosomes of KW1 and KW2, respectively. The strains contained four (KW1) and eight (KW2) plasmids, ranging in size and protein-coding genes (Table 1). Shintani et al. [22] showed that the plasmids of strains across the phylum *Firmicutes* range in size between 1.3 and 627 kb, with an average size of 39 kb. The plasmids in KW1 and KW2 have an average size of 48 kb and 35 kb, respectively. For further characterization of the plasmids, we determined the origin of replication and type of replicon using the web-tools Ori-Finder [23] and PlasmidFinder 2.1 [24, 25], respectively. Only four of the plasmids had replicons matching those in the PlasmidFinder 2.1 database (Table 1). A BLAST-search using the origin of replication sequence identified with Ori-Finder as the query showed that all replicons identified in this study are present in other lactic acid bacteria. Importantly, rep256 of the pSIP-plasmids is compatible with all the plasmids in KW1 and KW2, as demonstrated by the results described below. Analysis conducted with ResFinder 4.4.2 [25–27] revealed that none of the four plasmids from KW1 nor any of the eight plasmids from KW2 carry acquired antibiotic resistance genes.

**Table 1:** Size, number of annotated proteins, origin of replication, and type of replicon for plasmids in *L. pentosus* KW1 and KW2.

	Size (bp)	Number of annotated proteins	Origin of replication (nucleotide start - nucleotide end)^1)^	Type of origin of replication^2)^
**KW1**				
p1	40,436	49	4858–5382	Rep3
p2	77,291	81	1416–1785 / 75,766–77,284^3)^	-
p3	61,578	72	16,791–17,223	-
p4	13,269	19	10,195–11,149	-
**KW2**				
p1	52,982	37	25,152–25,325	Rep3
p2	16,730	13	12,463–12,742	-
p3	60,393	41	60046-431	-
p4	60,886	53	60,271–60,990	RepA_N
p5	27,312	23	26,025–26,873	-
p6	51,922	35	41,936–42,040	RepA_N
p7	6,340	4	5495–5907	-
p8	3,492	4	1-1175	-

^1)^ Determined using Ori-Finder

^2)^ Determined using Plasmid-Finder. Note that the type can only be determined for plasmids that occur in the PlasmidFinder database; -, non determined

^3)^ For this plasmid, Ori-Finder predicted two origins of replication

Both KW1 and KW2 encode the signature proteins Cas3 and Cas9 for type I and II CRISPR systems, respectively [28]. Interestingly, the seven spacer sequences present in the genomes of KW1 and KW2 are identical between the strains, all 31 nucleotides in length. A BLAST-search of the spacers resulted in zero hits to organisms other than KW1 and KW2. The proteins necessary for spacer acquisition, Cas1 and Cas2, are also encoded in both genomes. Both genomes contain two Cas1 encoding genes with 100% sequence similarity. The Cas2 amino acid sequences are also identical between the two strains. The Cas9 proteins encoded by KW1 and KW2 are highly divergent from the well-known Cas9 proteins encoded by *S. pyogenes* and *S. aureus*, in accordance with previous studies of lactobacilli [29].

### Detection of CPS production by KW1 and KW2

**Fig. 1:**
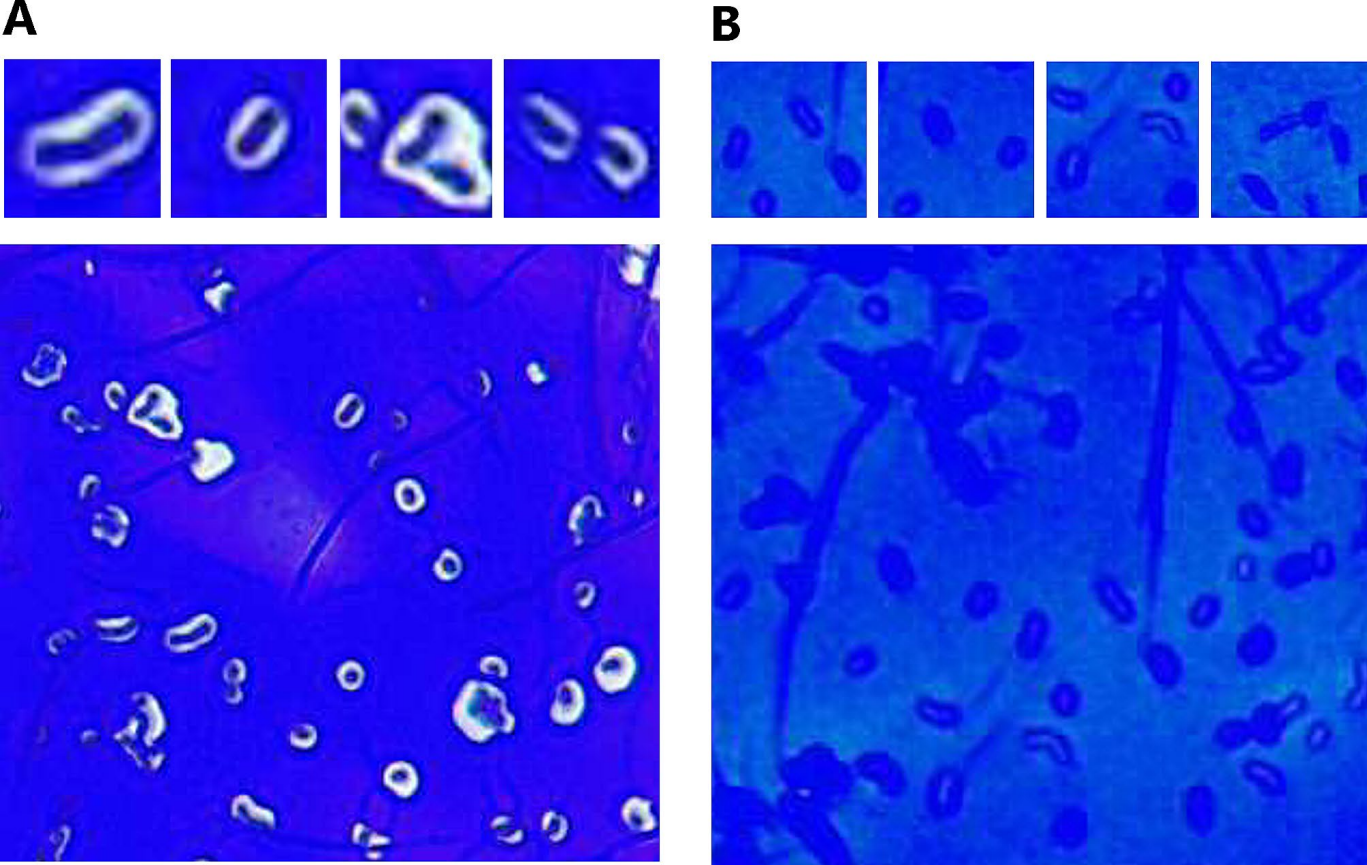
Negative staining of *L. pentosus* KW1 **(A)** and KW2 **(B)**. Capsular polysaccharides (CPS) were stained with nigrosine (white halo), and the bacteria were counter stained with crystal violet (violet)

We observed distinct morphological differences between the strains, with the pellet of KW1 being especially viscous after high-speed centrifugations. To further investigate these differences, negative staining was performed to visualize the presence of CPS (Fig. 1). Nigrosin was used as the negative stain, and crystal violet was used as the counter stain. Interestingly, the results indicate that KW1 produces CPS, seen as white halos surrounding the bacteria (Fig. 1A), while such halos are not visible for the stained KW2 cells (Fig. 1B).

### Secretome analysis of *L. pentosus* KW1 and KW2

The main components of the secretion machinery, i.e. the signal recognition particle (SRP) and SRP receptor (Ffh and FtsY respectively), the general chaperone trigger factor (Tf), SecA, SecB, SecE, SecG, SecY, YajC, YidC and PrsA were all found to be encoded in both genomes. Additionally, both strains contain a single copy of sortase A, which is necessary for cleavage and covalent anchoring of LPxTG-motif proteins to the cell wall. Both strains have three signal peptidase I (SPI) homologs and a single lipoprotein signal peptidase (SPII). Both genomes are predicted to encode a protein belonging to the A24 family peptidases, NGP02_15160 in KW1 and LPKW2_14390 in KW2, assumed to be involved in processing of prepilin-proteins. The prepilin-peptidases in KW1 and KW2 are identical, and share 65.9% identity with PulO from *L. plantarum* WCFS1.

Although heterologous anchoring sequences from *L. plantarum* WCFS1 proved functional in *L. pentosus* KW1 and KW2 (data not shown), we embarked on identifying and assessing new anchors homologous to each host strain. To do so, first, the secretome of *L. pentosus* KW1 and KW2 was predicted. All protein sequences were analyzed using SignalP6.0 to determine the presence of SPI (LysM-motif-, LPxTG-, WxL- and CTTM-containing proteins), SPII (lipoprotein) or SPIII (pilin) cleavage sites as well as proteins lacking a cleavage site but containing an N-terminal transmembrane (NTTM) anchor (Table 2). Sequences containing a signal peptide with a SPI site were further analyzed with SMART-EMBL to determine the presence of LysM- and WxL-motifs and with pSortb to determine the presence LPxTG cell wall binding-motifs. Analysis of the remaining proteins with a SPI-cleavage site using the TMHMM database enabled detection of possible C-terminal transmembrane (CTTM) anchors, resulting in a predicted 16 (KW1) and 11 (KW2) proteins containing a CTTM-anchor. The pSortb analysis revealed 14 and 12 sortase-anchored proteins in KW1 and KW2, respectively, and showed that the conserved sortase motif sequence was LPQTxE rather than LPxTG in both strains, which is also the consensus motif in *L. plantarum* WCFS1 [30], a close phylogenetic relative of *L. pentosus.* Similar to the LPxTG-motif, the WxL-motif, found in 11 (KW1) and 6 (KW2) proteins, is located at the C-terminus of the protein and mediates cell wall binding in gram-positive bacteria. However, while cell wall binding of LPxTG-proteins is covalent, the WxL-mediated binding is suggested to be noncovalent [31]. The WxL-domain was not used for antigen surface display in the present study. KW1 contains eight proteins containing a single LysM-domain, and one protein containing two LysM-domains. In KW2, six proteins contain one LysM-domain, and one protein contains two LysM-domains. All sequences predicted to have a SPII site, 67 and 60 in KW1 and KW2, respectively, were considered to be lipoproteins. The proteins predicted by SignalP 6.0 to be without a cleavage site were further analyzed with TMHMM to identify proteins corresponding to the structure of a NTTM-anchor. The analysis revealed the presence of 38 and 52 NTTM-anchors in KW1 and KW2, respectively. Analysis of the secretome revealed that both KW1 and KW2 have more SPI-proteins than SPII-proteins, while the total number of secreted proteins is higher in KW1 (227) than in KW2 (215) (Table 2). In addition, the KW1 and KW2 proteomes were predicted to contain two and three pilin proteins with an SPIII-cleavage site, respectively. All of the proteins with a SPIII cleavage site were annotated as hypothetical proteins, but through analysis with SMART-EMBL, the identical proteins NGP01_04215 and LPKW2_08865 were predicted to be involved in the methylation of a conserved phenylalanine residue found in the N-terminal region of pilins.

**Table 2:** The predicted secretome of *L. pentosus* KW1 and KW2. Additional proteins transcribed from genes located on plasmids are indicated in parentheses

Cleaved by		KW1	KW2
SPI	LysM	8 (1)	7 (1)
	LPxTG	14 (0)	12 (1)
	WxL	11 (0)	6 (1)
	C-terminal transmembrane proteins	16	11
	Secreted/ other anchor	70 (4)	65 (8)
SPII	Lipo	67 (1)	60 (3)
SPIII	Pilin	3 (0)	2 (0)
No cleavage site	N-terminal transmembrane proteins	38	52

### Characterization of growth rate and functionality of the pSIP vector system for inducible gene expression in *L. pentosus* KW1 and KW2

**Fig. 2:**
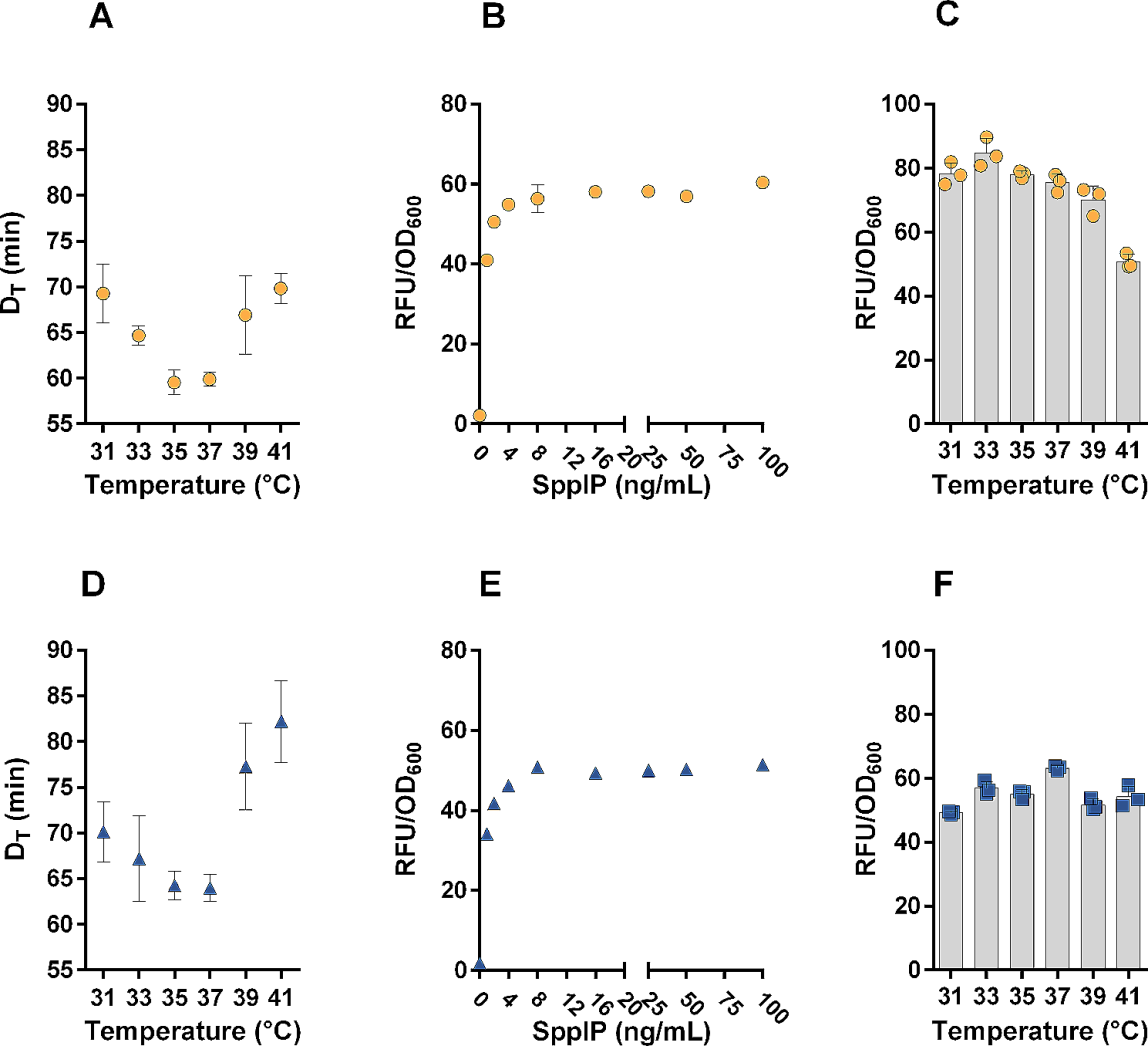
Characterization of the temperature-dependence of the growth rate **(A, D)**, the functionality of the inducible SIP-system **(B, E)** and the temperature dependence of heterologous protein production **(C, F)** in KW1 and KW2. The doubling time (D_T_) of wild type KW1 **(A)** and KW2 **(D)** was calculated during exponential growth. Recombinant KW1 and KW2 strains containing a vector for inducible production of the red fluorescent protein mCherry were used to determine how expression depends on the dose of the inducer, the SppIP peptide, at 37 °C **(B, E)**. The measured signals were normalized by dividing the fluorescent signal (RFU) by the OD_600_. The data shown refer to four hours after induction of the cultures. Note that almost no fluorescence was detected in cultures with no inducer. Also note that counting of the bacteria showed a similar relationship between the OD_600_ and the number of cells for KW1 and KW2 (see Materials and Methods section). Panels **(C, F)** show the temperature-dependency of mCherry-fluorescent signal at four hours after induction. All experiments **(A-F)** show the mean ± SD of two (**B, E**) or three (**A,C,D,F**) biological replicates

In order to determine the optimum temperature for growth, the strains were grown at temperatures from 31 to 41 °C (with 2 °C intervals), and the generation time was calculated in the exponential phase (formula 1). Figure 2A and D show doubling times in the range of 60–80 min at all tested temperatures with optimum temperatures for both strains between 35 and 37 °C.

The one-plasmid pSIP-system for inducible gene expression [32, 33] has been successfully used in many different lactic acid bacteria to express heterologous proteins [34–36], but Karlskås et al. [37] reported that the system was not functional in two tested strains of *L. pentosus.* As the performance of the pSIP expression system is highly host-dependent, we nevertheless assessed its functionality in KW1 and KW2 using red fluorescent mCherry as a reporter protein. Experiments with various dosages of the inducer peptide SppIP showed that the inducible P_*sppA*_ promoter driving expression of mCherry is tightly controlled and that mCherry expression occurred in an inducer dose-dependent manner up to SppIP concentrations of 8 ng/mL (Fig. 2B, E). Of note, this is a considerably lower maximum inducer concentration compared to what was found in a dose response assay performed with *L. plantarum* using the pSIP expression system [38].

After establishing the functionality of the pSIP-system, we used the mCherry strains to determine the temperature yielding maximal production of heterologous proteins (Fig. 2C and F). Interestingly, the temperature appeared to have a lesser effect on the production level in KW2 (Fig. 2F) compared to KW1 (Fig. 2C). The highest RFU/OD_600_ values, i.e. the optimum production temperature for the two strains, were found to be at 33 and 37 °C for KW1 and KW2, respectively. The production of mCherry was 29% higher in KW1 than in KW2 at their respective optimum temperatures. These experiments demonstrate, for the first time, the functionality of the pSIP-system in *L. pentosus*.

### Activation of antigen-presenting macrophages

**Fig. 3:**
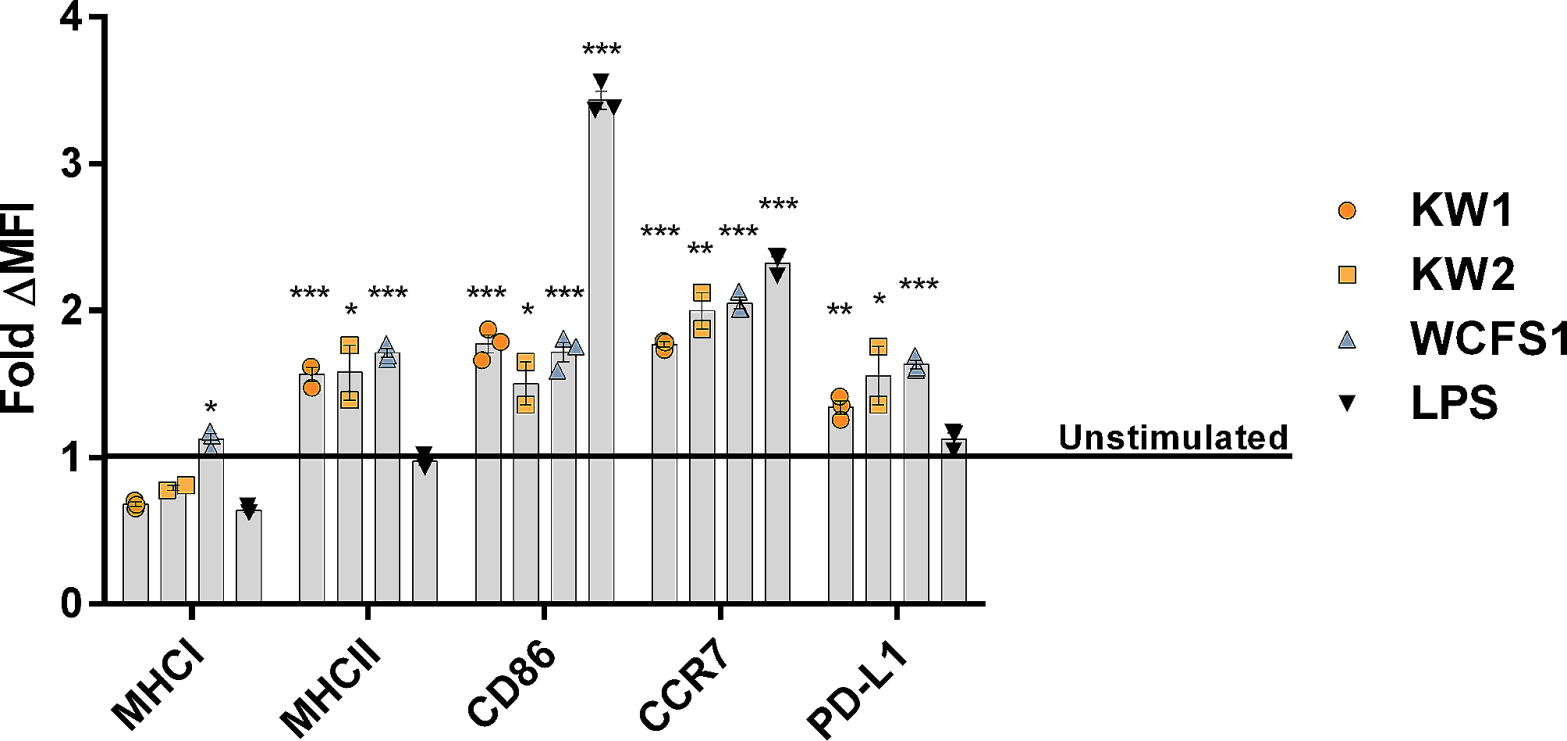
Stimulation of murine macrophages with UV-inactivated wild type KW1, KW2 and *L. plantarum* WCFS1 cells. LPS from *E. coli* was used as a positive control of stimulation. The macrophages (1 × 10^6^) were stimulated with 1 × 10^8^ UV-inactivated bacteria for 48 h, and the fold change in the presence of key surface proteins of antigen presenting cells were analyzed by flow cytometry. The data are presented as the mean of fold change in MFI between stimulated and unstimulated samples ± SEM. The MFI of the individual surface markers was found by using a single cell → mononuclear cell → Live → MFI of specific surface marker gating strategy. The mean percentages of live macrophages stimulated with KW1, KW2, WCFS1 and LPS were 98.4, 97.4, 97.0 and 92.7, respectively. The figure shows two (KW2) or three biological replicates. Pairwise two-tailed T-tests were performed between all stimulated samples and the unstimulated sample to test for statistical differences. *p < 0.05, **p < 0.01, ***p < 0.001

In order to assess the capacity of the novel *L. pentosus* strains to activate macrophages for antigen presentation, flow cytometry analysis was employed to examine the upregulation of relevant surface markers (Fig. 3). Before performing the analysis, we stimulated mouse macrophages with UV-inactivated wild type KW1, KW2 and *L. plantarum* WCFS1 for 48 h. A similar experiment with LPS from *E. coli* was included as a positive control. *L. plantarum* WCFS1, a well-known immune stimulating bacterium, was included to aid in the evaluation of the potency of the *L. pentosus* stimulation. The analysis revealed significant upregulation of MHCII, CD86, CCR7 (CD197) and PD-L1 (CD274) upon exposure of the macrophages to all tested strains. The fold increase in median fluorescent intensity (MFI) of MHCII and PD-L1 was markedly higher for cells stimulated with KW1, KW2 and WCFS1 compared to LPS-stimulated cells. The strains, nor LPS, did not stimulate expression of MHCI, which mainly displays peptides derived from the cell’s own proteins. There does not appear to be a pronounced difference in the ability to activate macrophages between KW1 and KW2, and the level of activation induced by the novel strains is comparable to that of *L. plantarum* WCFS1. For most markers, stimulation by the bacteria was similar to stimulation by LPS, except for CD86, which was clearly more upregulated in cells exposed to LPS.

Furthermore, we conducted macrophage-stimulation experiments with KW1, KW2 and *L. plantarum* WCFS1 harboring surface displayed antigens (more details below) which gave results similar to those obtained with the wild type strains (data not shown).

### Production and surface display of an *M. tuberculosis* hybrid antigen in *L. pentosus* KW1 and KW2

After confirming that the inducible pSIP-system is strictly regulated and functional in both strains, the expression system was used to express the H56 antigen [39], a mycobacterial hybrid antigen, translationally fused to signal peptides and anchoring motifs derived from the KW1 and KW2 genomes. As the source of the secretion and anchoring signals, we selected four proteins predicted to contain N-terminal transmembrane- (NTTM), lipoprotein- (Lipo), LPxTG -, and LysM –anchors for each strain. The NTTM-anchors were derived from NGP02_02010 (KW1) and LPKW2_12625 (KW2). The N-terminal, cytoplasmic part of the NTTM-anchors is positively charged and consists of 11 (KW1) and 43 (KW2) amino acids, while the length of the extracellular part of the NTTM-anchor, downstream of the 20-residue transmembrane region, was constructed to be of equal length (52 amino acid residues), similar to the length of the lipoprotein-anchor. For non-covalent attachment to the cell wall using a LysM-anchor, the antigen sequences were fused to the C-terminus of the full-length proteins NGP02_07240 (KW1) and LPKW2_06475 (KW2). The lipoproteins NGP02_02310 from KW1 and LPKW2_10785 from KW2 and the LPxTG-proteins NGP02_06930 from KW1 and LPKW2_06070 from KW2 share 100% sequence similarity. Thus, only one plasmid for production of Lipo-anchored H56 and one plasmid for LPxTG-anchored H56 was constructed and transformed to both strains. Accordingly, six plasmids with different secretion signals and anchors translationally fused to H56 were constructed (Table 3), yielding eight recombinant strains which were analyzed for growth and surface display of H56.

**Table 3:** Plasmids and strains used in this study

Strains	Description	Reference
*Lactiplantibacillus plantarum* WCFS1	Host strain	[30]
*Lactiplantibacillus pentosus* KW1	Host strain	This study
*Lactiplantibacillus pentosus* KW2	Host strain	This study
*Escherichia coli* Stellar	Subcloning strain	Takara Bio
KW1-pEV	*L. pentosus* KW1 harboring pEV	This study
KW1-Cyt-H56	*L. pentosus* KW1 harboring pSIP_CytH56	This study
KW1-NTTM-H56	*L. pentosus* KW1 harboring pSIP_KW1_NTTM_H56	This study
KW1-Lipo-H56	*L. pentosus* KW1 harboring pSIP_KW1-2_Lipo_H56	This study
KW1-LysM-H56	*L. pentosus* KW1 harboring pSIP_KW1_LysM_H56	This study
KW1-LPxTG-H56	*L. pentosus* KW1 harboring pSIP_KW1-2_LPxTG_H56	This study
KW2-pEV	*L. pentosus* KW2 harboring pEV	This study
KW2-Cyt-H56	*L. pentosus* KW2 harboring pSIP_CytH56	This study
KW2-NTTM-H56	*L. pentosus* KW2 harboring pSIP_KW2_NTTM_H56	This study
KW2-Lipo-H56	*L. pentosus* KW2 harboring pSIP_KW1-2_Lipo_H56	This study
KW2-LysM-H56	*L. pentosus* KW2 harboring pSIP_KW2_LysM_H56	This study
KW2-LPxTG-H56	*L. pentosus* KW2 harboring pSIP_KW1-2_LPxTG_H56	This study
**Plasmids**	**Description**	**Reference**
pEV	Ery^r^; 256_rep_; pSIP401 derivative; control plasmid (“empty vector”)	[68]
pSIP_1261_H56-DC	Ery^r^; 256_rep_; pSIP401 derivative containing the inducible P_*sppA*_ promoter fused to a gene construct encoding the lipoprotein anchor sequence from the gene *lp_1261* followed by the sequence encoding H56 and a dendritic cell binding (DC) peptide [69].	This study
pJET1.2_ESAT-6-Rv2660c	Amp^r^; pJET1.2 derivative containing a gene fragment encoding the fused *M. tuberculosis* derived antigens ESAT-6 and Rv2660c.	GenScript
pLp_1261AgE6-DC	Ery^r^; 256_rep_; pSIP401 derivative containing the inducible P_*sppA*_ promoter translationally fused to the lipoprotein anchor sequence derived from *lp_1261* followed by a sequence encoding AgE6 fused to a DC-peptide.	[18]
pSIP_KW1_NTTM_H56	Ery^r^; 256_rep_; pSIP401 derivative containing the inducible P_*sppA*_ promoter translationally fused to an N-terminal transmembrane anchor (NTTM) derived from *NGP02_02010* followed by the sequence encoding H56 and the DC-peptide.	This study
pSIP_KW2_NTTM_H56	Ery^r^; 256_rep_; pSIP401 derivative containing the inducible P_*sppA*_ promoter translationally fused to an N-terminal transmembrane anchor (NTTM) derived from *LPKW2_12625* followed by the sequence encoding H56 and the DC-peptide.	This study
pSIP_KW1-2_Lipo_H56	Ery^r^; 256_rep_; pSIP401 derivative containing the inducible P_*sppA*_ promoter translationally fused to the N-terminal signal peptide and Lipoprotein anchor derived from *NGP02_02310* from KW1 (identical to *LPKW2_10785* in KW2) followed by the sequence encoding H56 and the DC-peptide.	This study
pSIP_KW1_LysM_H56	Ery^r^; 256_rep_; pSIP401 derivative containing the inducible P_*sppA*_ promoter translationally fused to the N-terminal signal peptide and LysM anchor derived from the gene *NGP02_07240* followed by the sequence encoding H56 and the DC-peptide.	This study
pSIP_KW2_LysM_H56	Ery^r^; 256_rep_; pSIP401 derivative containing the inducible P_*sppA*_ promoter translationally fused to the N-terminal signal peptide and LysM anchor derived from the gene *LPKW2_06475* followed by the sequence encoding H56 and the DC-peptide.	This study
pSIP_3050_H56_cwa3001	Ery^r^; 256_rep_; pSIP_3050_H1_cwa3001 derivative containing the inducible P_*sppA*_ promoter translationally fused to the N-terminal signal peptide derived from the gene *lp_3050* followed by the sequence encoding H56 and a C-terminal LPxTG anchor from *lp_3001.*	This study
pSIP_3050_H1_cwa3001	Ery^r^; 256_rep_; pLp_3050DC-AgE6cwa2 [18] derivative containing the inducible P_*sppA*_ promoter translationally fused to the N-terminal signal peptide derived from *lp_3050* followed by the sequence encoding H1 and a C-terminal LPxTG anchor from *lp_3001.*	Unpublished
pSIP_KW1-2_LPxTG_H56	Ery^r^; 256_rep_; pSIP401 derivative containing the inducible P_*sppA*_ promoter translationally fused to the N-terminal signal peptide and LPxTG anchor derived from *NGP02_06930* (identical to *KW2_01306* in KW2) followed by the sequence encoding H56.	This study
pSIP_CytH56	Ery^r^; 256_rep_; pSIP401 derivative containing the inducible P_*sppA*_ promoter translationally fused to H56 and the DC-peptide.	This study
pSIP403_mCherry	Ery^r^; 256_rep_; pSIP403 derivative, encoding red fluorescent protein mCherry under control of the inducible P_*sppA*_ promoter.	[16]

**Fig. 4:**
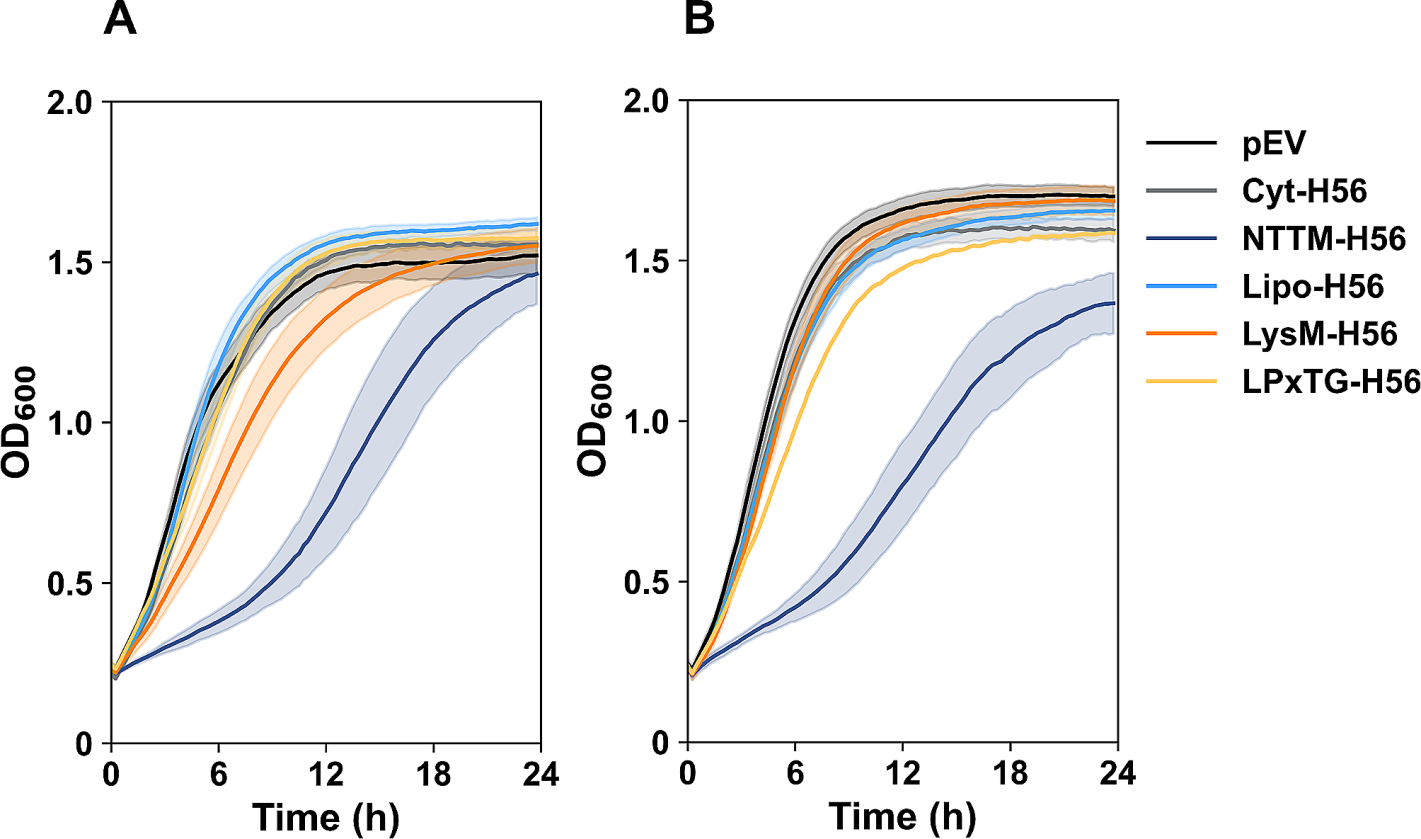
Growth curves for recombinant KW1 **(A)** and KW2 **(B)** strains. Induced cultures of *L. pentosus* KW1 **(A)** or KW2 **(B)** carrying various expression plasmids were transferred to a sterile 96-well plate directly after induction (time point 0 h), and the OD_600_ of the cultures was continuously measured every 15 min for 24 h at 37 °C. The data shown are a mean of three biological replicates; standard deviations are indicated by shading. In the absence of induction, all strains showed the same growth curves, similar to those of induced pEV (data not shown)

After transformation of the plasmids to *L. pentosus* KW1 and KW2, the growth of the induced recombinant strains was analyzed (Fig. 4). The strains producing Lipo-H56, LysM-H56 or LPxTG-H56 grew similar to the strain carrying the empty vector plasmid (pEV) and the strain producing cytoplasmic H56 (Cyt-H56). On the other hand, both strains expressing H56 with an NTTM-anchor showed markedly reduced growth rate. The rapid growth of KW1-LPxTG-H56 and KW2-LPxTG-H56 is contrary to what has been observed previously in other *Lactobacillales* producing LPxTG-anchored heterologous proteins [34].

**Fig. 5:**
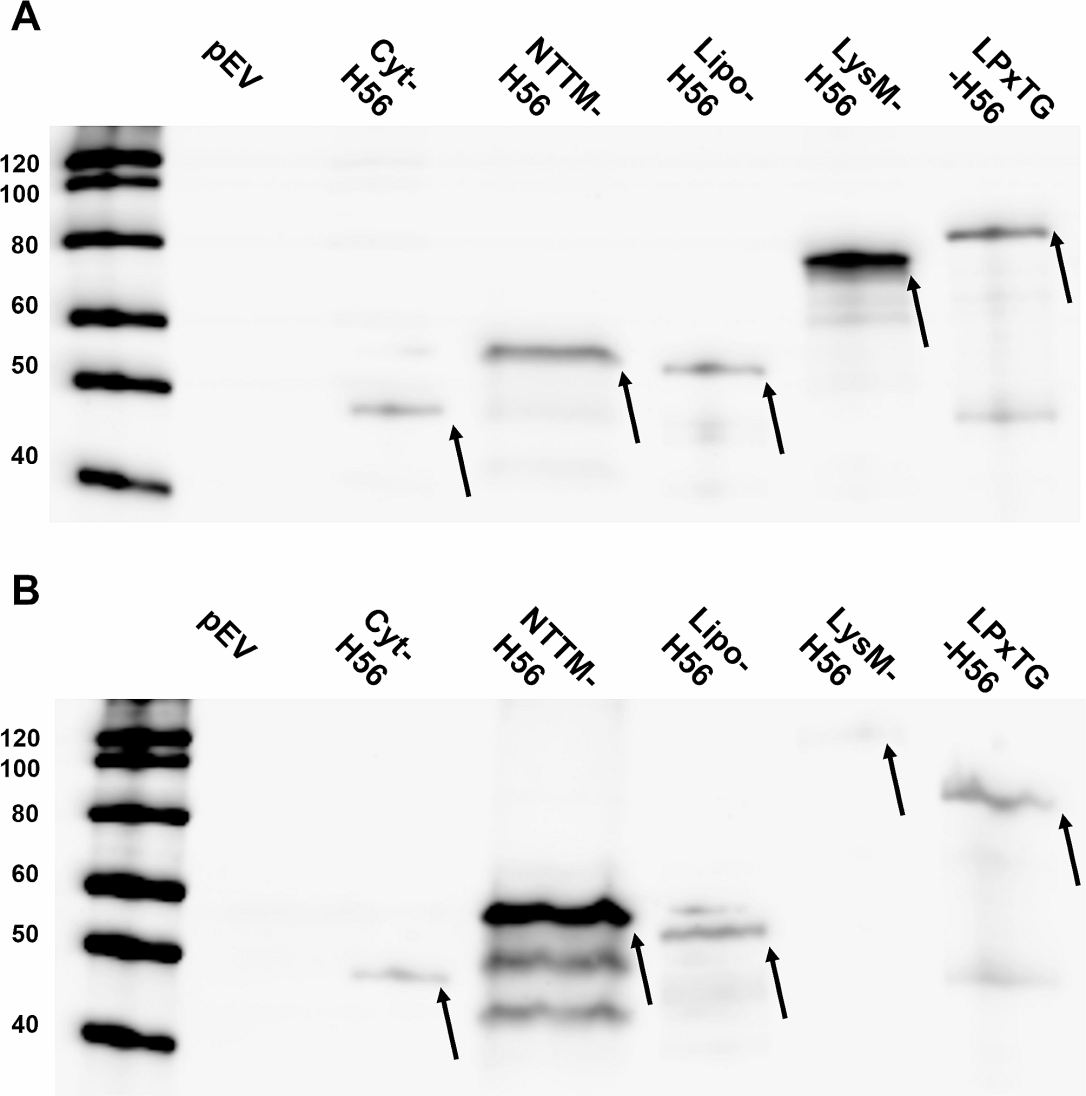
Western blot analysis of cell-free extracts from recombinant KW1 **(A)** and KW2 **(B)** strains producing H56 fused to various anchoring sequences. About 0.8 µg of protein was loaded for the KW1 strains, while 1.6 µg was loaded of most KW2 strains, except the lanes labeled NTTM-H56 and LysM-H56 which contain 2.4 µg of protein. Well 1: Magic Marker, well 2: pEV, well 3: Cyt-H56 (predicted protein size ~ 50 kDa, both strains), well 4: NTTM-H56 (KW1: 60 kDa, KW2: ~65 kDa), well 5: Lipo-H56 (~ 58 kDa, both strains), well 6: LysM-H56 (KW1: 60 kDa, KW2: ~86 kDa), well 7: LPxTG-H56 (~ 78 kDa, both strains). The arrows indicate the fusion protein

Western blot analyses of the H56-producing strains (Fig. 5) revealed protein bands at positions corresponding well with the expected protein sizes for all five protein variants, while no band was detected in the pEV negative control. Differences in band intensity between KW1 and KW2 varied, depending on which protein variant was expressed. The production of the LysM-H56 protein by KW2 appears to be particularly low.

**Fig. 6:**
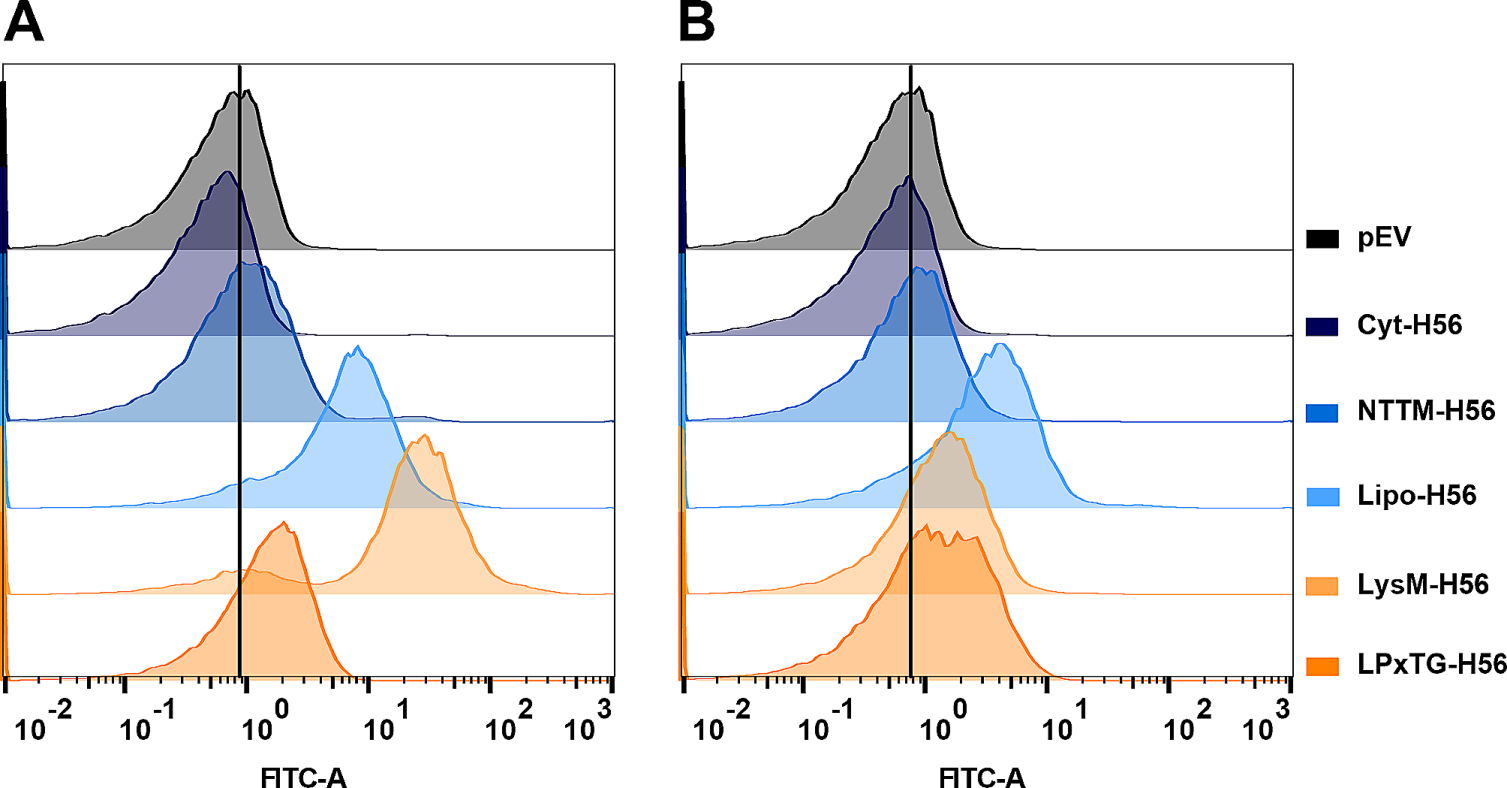
Flow cytometry analysis of the presence of the H56 antigen on the surface of recombinant *L. pentosus* KW1 **(A)** and KW2 **(B)** strains carrying various expression vectors. The experiment was repeated three times. The data presented are from one representative experiment

Subsequently, we performed a flow cytometry analysis to verify surface exposure of the antigen. As expected, no fluorescent signal could be detected with bacteria harboring either of the control plasmids (empty vector, or a vector encoding for non-secreted H56; Fig. 6). Analysis of the other recombinant strains showed that H56 was successfully anchored to and exposed on the surface of the producing bacterium through Lipoprotein-, LysM- and LPxTG-anchoring. It is worth noting that the LysM signal obtained with KW2 is much weaker compared to KW1, which aligns well with the large difference in protein production that is suggested by the Western blot of Fig. 5. While H56 with an N-terminal transmembrane anchor (NTTM-H56) was detected in both strains in the Western blot (Fig. 5), the shift obtained in flow cytometry was weak (Fig. 6) suggesting that the amount of surface displayed antigen was minute. It may thus seem that translocation of this protein is problematic, in accordance with the observations that the strains producing this H56 variant show growth defects upon induction (Fig. 4) and that protein degradation occurs (visible for KW2 in the Western blot; Fig. 5B).

## Discussion

In this study we present the genome sequences of *Lactiplantibacillus pentosus* KW1 and KW2, isolated from green olives, and we assess the potential of these strains as delivery vehicles. The KW1 genome consists of a circular chromosome and four plasmids, while KW2 harbors eight plasmids. The number of plasmids in *L. pentosus* is known to be highly variable, ranging from zero plasmids in *L. pentosus* KCA1 to three, five and six plasmids in *L. pentosus* LTJ12, F03 and 9D3, respectively [40–43]. The G + C contents of the two the strains were nearly identical (46.4% and 46.5%). In accordance with our results, GenBank reports that the median G + C content of all deposited *L. pentosus* strains (84 strains) is 46%. Although plasmid-free strains may be better for biotechnological purposes, the original plasmids harbored by KW1 and KW2 did not hamper the use of the pSIP-plasmids for protein expression. Similarly, *L. plantarum* WCFS1 is widely used for recombinant protein expression using the pSIP system, while carrying three native plasmids [30]. Because the native plasmids of KW1 and KW2 are compatible with the rep256 from the pSIP-plasmid, the replicons of these plasmids could be employed for development of novel vectors for lactic acid bacteria.

The large genomes of these bacteria indicates a nomadic lifestyle, as more specialized strains tend to have markedly smaller genomes [44]. Concurrently, EPS production is considered to facilitate niche adaption by promoting aggregation, surface attachment and biofilm formation. A gene cluster associated with EPS-production present in *L. pentosus* L33 and SLC13 [7, 45], was also identified in the chromosomes of KW1 and KW2, which indicates that both strains are EPS producers. Moreover, staining experiments showed that KW1 appears to produce CPS, which might explain the observed viscosity of the KW1 cells when pelleted. Production of EPS/CPS could affect protein production and surface-display, although, in the present study, protein production was assessed in the exponential phase, when production of EPS/CPS is less likely. A deeper genomic analysis of the EPS- producing potential of KW1 and KW2 and further studies of how this potentially affects protein display would be of interest.

Both *L. pentosus* genomes encode all key proteins of the secretion machinery and the sortase recognition motif is LPQTxE. The assembled KW1 genome encodes 228 more proteins than the KW2 genome, predominantly proteins without a signal peptide. KW1 also encodes more proteins destined for the extracellular environment (Table 2). In particular, the numbers of proteins harboring a WxL-motif are notably higher in KW1. Bacterial species encoding WxL-proteins are mostly limited to gut commensal species, and WxL proteins are considered to be important for degradation and utilization of oligo- and polysaccharides [46]. For the chromosomally encoded proteins, the percentage of secreted proteins appears to be considerably lower in KW1 and KW2 (6.9% and 6.3%) compared to e.g. *L. pentosus* KCA1 (9.3%) [41] and *L. plantarum* WCFS1 (7.3%) [30]. For plasmid-encoded proteins, the fraction of secreted or surface displayed proteins is even lower (2.7 in KW1 and 3.8% in KW2). Since secreted proteins are important for nutrient uptake and bacterial fitness [47], these low fractions of secreted proteins could indicate limitations in the ecological versatility of *L. pentosus* KW1 and KW2.

Initial studies with the inducible pSIP-system and the mCherry reporter protein showed that the system is tightly regulated, and sensitive to the concentration of inducer peptide. Since high levels of heterologous gene expression can lead to stress, access to a well-regulated expression system may be advantageous for biotechnological applications. Upon the addition of only 1 ng/mL of SppIP, mCherry production reached 68% and 66% of maximum production in KW1 and KW2, respectively. Differences in mCherry levels were negligible at inducer concentrations between 8 and 100 ng/mL, indicating that addition of 8 ng/mL SppIP is sufficient for achieving maximum protein production. Previous studies with *L. sakei* and *L. plantarum* concluded that inducer levels of at least 10 ng/mL and 25 ng/mL SppIP were needed for reaching maximum protein production, respectively [32, 38]. In *L. plantarum* the system is normally used with inducer concentration between 25 and 100 ng/mL [16, 37, 48, 49]. In an application setting, a strain that can be fully induced with a low peptide concentration is beneficial due to the cost of the peptides.

The experiments with mCherry also showed that protein production levels are similar in the 31–41^o^C range, although KW1 showed a relatively clear optimum at the lower tested temperatures. KW1 may have a lower optimal production temperature than KW2 due to generally higher production of mCherry, leading to more stress, which could have more impact at higher temperatures.

Several lactic acid bacteria, such as *L. plantarum* and *Lactococcus lactis, L. casei* and *Lactobacillus helveticus*, have been shown to activate the immune system although the impact varies between species [50]. Our studies with mouse macrophages (Fig. 3) show that the surface expression of MHC-II, CD86, CCR7 and PD-L1 is significantly upregulated in macrophages exposed to *L. pentosus* KW1 or KW2. MHC-II, CD86 and PD-L1 are important surface proteins linked to increased T cell proliferation in in vitro studies with human cells [51, 52], while CCR7 is important for cell migration of the cells involved in the adaptive immune system [53]. In accordance with these observations, You et al. [8] showed that stimulation of murine macrophages with purified EPS from *L. pentosus* LZ-R-17 results in a potent immune response *in vitro.* Despite apparent differences in EPS production, we did not observe differences between KW1 and KW2 in the macrophage stimulation assay. This is likely because the bacteria used for macrophage stimulation were harvested at the exponential phase, at which time EPS production is limited [54]. All in all, the macrophage stimulation assay demonstrates that KW1 and KW2 may have adjuvant effects that could strengthen their suitability as delivery vehicles for immunization.

*L. pentosus* is a relatively unexplored strain for antigen delivery. Based on the encouraging results and strain characteristics discussed above, we further verified the potential of strains KW1 and KW2 by constructing recombinant strains for production and surface display of the tuberculosis hybrid antigen H56. For most recombinant strains, production of H56 did hardly affect growth which is unusual for surface display of heterologous proteins (e.g. see [34, 55]) and of major importance, since this allows easy production of sufficient amounts of antigen-carrying bacterial cells. Notably, the performance of the strains depended on both the type of H56 construct and the strain. For example, while growth defects generally were limited, both strains showed evident growth reduction when producing NTTM-H56. It has been shown previously that overproduction of transmembrane proteins, may lead to decreased fitness and poor growth [56, 57]. Furthermore, the western blot analysis showed that production of NTTM-H56 is high compared to the other recombinant proteins, especially in KW2. Poor growth and high heterologous protein production are often correlated [34, 55, 58], and may be caused by secretion stress. However, flow cytometric analyses indicated that NTTM-H56 was hardly displayed on the bacterial surface. The latter could perhaps be attributed to NTTM-anchoring resulting in a cell wall embedded H56, although the external part of the NTTM-anchor was designed to be of similar length as the Lipo-anchor and should thus be detectable. It seems more likely that the lack of detection of NTTM-H56 is due to the problems discussed above and that this anchor, thus, is not a good choice for antigen display in *L. pentosus* KW1 and KW2.

As to differences between the strains, the production of LysM-H56 was notably different in KW1 and KW2. In KW1 LysM-H56 showed a clearly stronger protein band (note the different sample amounts used in the western blot analysis) and flow cytometric analysis showed a stronger signal for KW1 producing LysM-H56 compared to KW2 producing LysM-H56. The differences in protein production may be attributed in part to the N-terminal signal peptide, which differed between the KW1 and the KW2 constructed, since it was co-selected with the LysM domains derived from KW1 and KW2, respectively. The signal peptide is an important determinant of the secretion efficiency of heterologous proteins [59, 60].

Clear signals indicative of surface-display were observed in the flow cytometric analysis of strains carrying Lipo-H56 and LPxTG-H56. For Lipo-H56 a slightly stronger signal was detected with KW1 compared to KW2, even though in this case, the strains carried identical plasmids, which could indicate that KW1 on average is a somewhat better protein producer, as also suggested by the experiments with mCherry. Based on the growth curves, the western blots and flow cytometry, strain KW1 carrying LysM-H56 seems to have highest potential as a delivery vehicle for surface-displayed H56.

## Conclusion

Our results show that *L. pentosus* KW1 and KW2 are promising strains for vaccine delivery with, considering observed differences in EPS formation, and possibly differing adjuvant properties. In addition, this study presents the first homologous tools (signal peptides, anchors) that can be used in the generation of recombinant strains for antigen display by these strains. When using the inducible pSIP expression vector system, the strains are capable of producing and surface-anchoring a complex hybrid antigen, with, depending on the anchor sequence, sometimes only minimal growth defects. Thus, the present study demonstrates significant potential of *L. pentosus* KW1 and KW2 as new species for both antigen delivery and production of heterologous proteins.

## Materials and methods

### Bacterial strains, plasmids and growth conditions

The bacterial strains and plasmids used in this study are listed in Table 3. *Lactobacillus* selection media (LBS) was used for strain isolation of *L. pentosus* KW1 and KW2 from olives. *Escherichia coli* was cultivated in BHI medium (Oxoid Ltd., Basingstoke, United Kingdom) at 37 °C with agitation. When necessary, erythromycin was added to a final concentration of 200 µg/mL. Unless stated otherwise, *L. plantarum* and *L. pentosus* were cultured in MRS broth (Oxoid Ltd) without agitation at 37 °C. When necessary, erythromycin was added to a final concentration of 10 µg/mL. For plates, liquid BHI, MRS and LBS medium were solidified by addition of 1.5% (w/v) agar.

### Strain isolation

Green olives were diced into ~ 2 mm^2^ pieces and transferred to a tube containing 10 mL LBS media and incubated at 37 °C for 2 days. The cultures with visible growth were spread undiluted on LBS agar dishes for single colony isolation. The 16 S rRNA amplicon was obtained by running colony PCR using specific primers (Additional file 1: Table S1) on the colonies that appeared, and the obtained PCR fragments were subsequently purified, using the NucleoSpin® Gel and PCR Clean-up purification kit (Macherey-Nagel, Düren, Germany), and sequenced (Eurofins GATC Biotech, Konstanz, Germany). The sequencing identified two colonies (KW1 and KW2) as *Lactiplantibacillus pentosus*, and these strains were subjected to whole genome sequencing.

### Whole genome sequencing and annotation

Genomic DNA (gDNA) from *L. pentosus* KW1 and KW2 was used as template for sequencing. High molecular weight gDNA was isolated using the Nanobind HMW DNA Extraction kit (Circulomics Inc, Baltimore, MD). The extracted gDNA was prepared for sequencing using the Rapid Sequencing kit (SQK-RAD004), according to the manufacturer’s protocol (Oxford Nanopore Technologies, Oxford, UK). The libraries were loaded onto Nanopore R9.4.1 flow cells, and sequenced using the MinION Mk1B device with MinKNOW v3.6.5 from Oxford Nanopore Technologies. The reads were base called using Guppy v3.2.10 in ‘fast’ mode. This generated 4.11 Gb and 2.47 Gb of data for KW1 and KW2, respectively. Low quality base calls and short sequences (length < 1000 kb) were removed using Filtlong v0.2.0 (https://github.com/rrwick/Filtlong). The filtered sequences were assembled into contigs using Flye [61] v2.7 (https://github.com/fenderglass/Flye). After initial polishing with Racon [62] v1.4.14 (https://github.com/isovic/racon), the contigs were further polished with Medaka v1.1.3 (https://github.com/nanoporetech/medaka). The contigs were quality-checked using BUSCO [63] v4.1.4, before proceeding with gene-prediction using PROKKA [64] v.1.13. The whole genome sequences of *L. pentosus* KW1 and KW2 have been uploaded to the NCBI GenBank (https://www.ncbi.nlm.nih.gov/genbank/) depository under accession numbers PRJNA850900 and PRJNA850901, respectively.

The subcellular location of the proteins was predicted by importing the complete list of amino acid sequences from each strain into the SignalP6.0 database (https://services.healthtech.dtu.dk/service.php?SignalP) [65]. This generated a list of proteins divided into four categories: no signal peptidase cleavage site, signal peptidase I (SPI) cleavage site, signal peptidase II (SPII) cleavage site or with a signal peptidase III cleavage site (pilin-like signal peptide). Amino acid sequences with a SPII cleavage site are predicted to be membrane-anchored lipoproteins. All the amino acid sequences with a SPI cleavage site were further analyzed with the SMART-EMBL (http://smart.embl-heidelberg.de/) and pSortb (https://www.psort.org/psortb/) tools to identify potential LysM- and WxL- and LPxTG-domains. Amino acid sequences with an SPI cleavage site without a LysM-, WxL- or LPxTG-domain were analyzed for transmembrane helices using TMHMM 2.0 (https://services.healthtech.dtu.dk/service.php?TMHMM-2.0). Proteins with SPI signal peptide and a predicted outside-inside orientation separated by one transmembrane region (TMR) and the absence of a LPxTG domain were assumed to be C-terminal transmembrane anchors (CTTM).

To identify proteins with an N-terminal transmembrane anchor (NTTM), the protein sequences without a predicted signal peptidase cleavage site underwent TMHMM-analysis. All proteins with exactly one TMR preceding an intracellular N-terminal and followed by an extracellular C-terminal were predicted to be anchored to the cell membrane through an NTTM. The remaining proteins in the no cleavage site data set, with either zero or more than one TMR, were predicted to be cytoplasmic or intramembrane proteins, respectively.

### Negative staining for detection of capsular polysaccharides

Cultures of *L. pentosus* KW1 and KW2 were grown to the stationary phase to allow initiation of EPS synthesis. One inoculating loop of 1% (w/v) nigrosin (Sigma-Aldrich, Saint-Louis, MO) and one loop of the culture were mounted onto an object glass and mixed well. The sample was evenly distributed into a thin, even coating using a second object glass to slide and smear the sample. The preparate was air dried and subsequently flooded with 1% (w/v) crystal violet (Sigma-Aldrich). Nigrosin-stained CPS will not be counterstained with crystal violet, leaving a white halo around the violet cell. Excess of crystal violet was poured off the object glass, and the sample was air-dried. The sample was analyzed using a light microscope (Leica, Wetzlar, Germany) at 400x magnification, using bright field settings.

### Analysis of the optimum temperature for growth

Cultures of wild type *L. pentosus* KW1 and KW2 were incubated at three different temperatures overnight (32 °C, 36 and 40 °C). The overnight cultures were diluted to an OD_600_ of ~ 0.15, and incubated at 32 °C, 36 and 40 °C for 1.5 h to reach the exponential phase. When the cultures had reached the exponential phase, 200 µL portions of each culture were transferred to sterile 96-well plates (ThermoFisher, Waltham, MA; two plates per culture) and incubated in separate ThermoMixers (Eppendorf, Hamburg, Germany) at different temperatures, without shaking. Plates with the culture that had been pre-incubated at 32 °C were placed at 31 and 33 °C, plates with the culture that had been pre-incubated at 36 °C were placed at 35 and 37 °C, while plates with the culture that had been pre-incubated at 40 °C were placed at 39 and 41 °C. The OD_600_ of the cultures was measured every hour for five hours in a Varioskan Lux Reader (ThermoFisher Scientific), starting (OD_start_) after transfer of the culture to the plates. The growth rate was calculated from the exponential part of the resulting growth curve, as the doubling time, using the formula ${D}_{T}=\frac{T}{3.3\text{log}\left(\frac{ODstop}{ODstart}\right)}$ (formula 1), were T equals the total time between two OD_600_-mesaurements at selected time points (OD_start_ and OD_stop_) in the exponential phase. The experiment was performed with biological triplicates and two technical replicates.

### Analysis of optimum temperature for heterologous protein production

Overnight cultures of *L. pentosus* KW1 and KW2 harboring pSIP403_mCherry (Table 3), were diluted to OD_600_ ~ 0.15 and incubated at 32 °C, 36 or 40 °C, similar to the procedure for the growth optimum analysis. At an OD_600_ ~ 0.30 ± 0.03, the cultures were induced with 25 ng/mL pheromone peptide (SppIP) [66], (CASLO ApS, Lyngby, Denmark), and 200 µL portions of the culture were transferred to sterile 96-well plates (ThermoFisher). The plates were incubated at specific temperatures (31–41 °C) in separate ThermoMixers (Eppendorf), as in the growth optimum analysis, and the OD_600_ and fluorescence (excitation 587 nm, emission 620 nm) were measured simultaneously every hour for five hours in a Varioskan Lux Reader (ThermoFisher Scientific). The OD_600_ measurements showed that, at all temperatures, the cells were in the exponential phase at four hours after induction, i.e., the time point for which data are reported in Fig. 2.

### Dose-response assay of the inducer peptide

A dose-response assay was performed to investigate the sensitivity of the pSIP-inducible expression system using mCherry as a reporter protein. Overnight cultures of *L. pentosus* KW1 and KW2 harboring pSIP403_mCherry were incubated at 37 °C and induced as described in the previous section. The pheromone concentration was varied from 0 to 100 ng/mL and the fluorescence and OD_600_ were measured concomitantly in a Varioskan Lux Reader (ThermoFisher Scientific) four hours after induction.

### Analysis of immune cell activation

To analyze immune cell activation properties of the isolated strains, macrophages were stimulated with UV-inactivated wild type *L. pentosus* KW1 and KW2 and *L. plantarum* WCFS1 at a multiplicity of infection of 1:100 for 48 h. Bacteria were counted in a Bürker chamber to prepare a dose of 10^8^ bacterial cells. Of note, the counting showed that similar concentrations of KW1 and KW2 cells, which were harvested in the exponential phase, give similar OD_600_ values. The mouse macrophage J774A.1 cell line (DSMZ, Braunschweig, Germany) was cultured in high glucose DMEM medium supplemented with 10% fetal bovine serum (FBS), 2 mM L-glutamine (all from ThermoFisher) and 1% penicillin/streptomycin in T-25 cell culture flasks (Sarstedt, Nümbrecht, Germany). The cells were maintained in a humidified incubator at 37 °C and 5% CO_2_ and passaged at a confluency of ~ 80%. Macrophages incubated with 5 µg/mL LPS from *E. coli* 026:B6 (00-4976-93, ThermoFisher) instead of bacteria were included as a positive control. After the 48-hour incubation, the bacteria were removed by washing of the adherent cells. The macrophages were detached from the plastic using a 2-position size small cell scraper (Sarstedt), transferred to a 15 mL tube, and centrifuged for 5 min at 125 g at 4 °C. The cells were resuspended in 200 µL PBS (ThermoFisher Scientific) and transferred to a 96 U-well plate.

First, the cells were stained with the LIVE/DEAD™ Fixable Violet Dead Cell stain Kit, diluted 1:1000 (ThermoFisher Scientific) and incubated at room temperature for 30 min, protected from light. The LIVE/DEAD stain was washed off with PBS. Subsequently, the cells were mixed with a 1:100 dilution of FC block (Biolegend, San Diego, CA) and incubated for 10 min before staining with antibodies targeting specific antigen presenting cell-markers. An antibody cocktail containing CD197-Brilliant Violet (Biolegend) and CD86-PE-Cy7, MHCI-FITC, MHCII-NovaFluor™ Blue 610-70 S, CD274-NovaFluor Yellow 690 (all from eBioscience/ThermoFisher Scientific), in a 1:50 dilution in PBS, was used to stain the cells. The cells were incubated with the cocktail for 20 min at 4 °C, and subsequently washed three times with PBS/1% BSA, fixed in 100 µL IC Fixation buffer (ThermoFisher Scientific) for 10 min before the fixation buffer was washed away and 100 µL PBS/1% BSA was added to each well. The plates were stored at 4 °C in the dark until analysis. The analysis was performed on a Cytoflex LX flow cytometer (Beckman Coulter, Brea, CA). Data were analyzed using the Kaluza Software (Beckman Coulter). A pairwise two-tailed T-test was performed between unstimulated cells and all stimulations using the GraphPad Prism 6 software.

### Plasmid construction

All plasmids and their sources are listed in Table 3. Gene fragments encoding selected N-terminal anchors (Lipoprotein-, N-terminal transmembrane- and LysM domain-anchor; see Results section) were inserted into the pSIP_1261_H56-DC vector. pSIP_1261_H56-DC was constructed by *Kpn*I/*Hind*III (New England Biolabs (NEB), Ipswich, MA) digestion of pJET1.2_ESAT-6-Rv2660c and the 554 bp ESAT-6-Rv2660c fragment was inserted in the backbone of *Kpn*I/*Hind*III digested pLp_1261AgE6-DC, yielding a plasmid encoding the H56 antigen (Ag85B-ESAT-6-Rv2660c) called pSIP_1261_H56-DC (see [39] for details on the H56 fusion antigen). The N-terminal anchor sequences were amplified with specific InFusion primers (Additional file 1: Table S1) and genomic DNA from *L. pentosus* KW1 and KW2 as the template, and subsequently cloned into the *Nde*I/*Sal*I (NEB) linearized pSIP_1261_H56-DC vector using the InFusion HD Cloning kit (Takara Bio, Kusatu, Japan). The Lipoprotein-anchor was derived from proteins that are identical in KW1 and KW2. These constructions yielded the expression plasmids pSIP_KW1_NTTM_H56, pSIP_KW2_NTTM_H56, pSIP_KW1-2_Lipo_H56, pSIP_KW1_LysM_H56 and pSIP_KW2_LysM_H56.

Constructs for expression of H56 with a C-terminal LPxTG-anchor and the associated N-terminal SPI signal peptide were generated using the pSIP_3050_H56_cwa3001 vector as a starting point. The pSIP_3050_H56_cwa3001 vector was constructed by amplification of ESAT-6-Rv2660c with InFusion primers 1643_H56_F/1643_H56_R (Additional file 1: Table S1). The amplicon was cloned into *Kpn*I/*Mlu*I (NEB) digested pSIP_3050_H1_cwa3001 using the InFusion HD Cloning kit (Takara Bio), yielding pSIP_3050_H56_cwa3001. Subsequently, the C-terminal LPxTG-anchor was amplified with specific InFusion primers (Additional file 1: Table S1) with genomic DNA from *L. pentosus* KW1 (identical sequences in KW1 and KW2) as the template and InFusion-cloned into the *Mlu*I/*Hind*III linearized pSIP_3050_H56_cwa3001 vector. After verification of successful insertion of the LPxTG-anchor, the N-terminal signal peptide from the same gene as the LPxTG-anchor originated from, was amplified with specific InFusion primers (Additional file 1: Table S1). The plasmid containing the newly inserted LPxTG-anchor (identical for KW1 and KW2) was linearized with *Nde*I and *Sal*I, thereby enabling insertion of the associated signal peptide (also identical for KW1 and KW2), yielding the final plasmid pSIP_KW1-2_LPxTG_H56.

pSIP_CytH56 was constructed by amplifying the H56 antigen with specific InFusion primers (Additional file 1: Table S1), using pSIP_KW1-2_Lipo_H56 as a template, and by cloning amplified H56 into a *Nde*I/*Hind*III linearized pSIP_KW1-2_Lipo_H56 vector. All constructed plasmids were verified by sequencing prior to transformation into the *L. pentosus* strains, following the protocol described in Aukrust, Brurberg and Nes [67].

### Western blot analysis

The bacterial cells were harvested, lysed and loaded onto an SDS-PAGE gel as previously described [16]. The Bradford assay (Bio-Rad Laboratories, Hercules, CA) was used to determine the protein concentration of the cell free lysates. Approximately 0.8 µg crude protein extract was loaded onto the SDS-PAGE gel. After electrophoresis, proteins were blotted onto a nitrocellulose membrane using an iBlot™ Transfer Device (Invitrogen, Waltham, MA). Following the protein transfer, the SNAP i.d. 2.0 kit (Sigma-Aldrich) was used for antibody hybridization to the H56 antigen according to the manufacturer’s protocol. The mouse monoclonal antibody anti-ESAT-6 (ab26246, Abcam Inc, Cambridge, United Kingdom) primary antibody was diluted 1:2 000, and the secondary antibody m-IgGκ BP-HRP (Santa Cruz Biotechnology, Dallas, TX) was diluted 1:15 000. The proteins were visualized using the SuperSignal West Pico PLUS Chemiluminescent substrate (ThermoFisher Scientific) and the signals were imaged with an Azure c400 system (Azure biosystems, Dublin, CA).

### Detection of surface displayed H56 antigen on *L. pentosus*

Bacterial cultures were grown and induced similar to the western blot samples. Cells from approximately 500 µL of culture were harvested 4 h after induction and washed once with PBS (8 000 g, 3 min, RT). The pellet was resuspended in a 1:333 dilution of the primary mouse monoclonal antibody anti-ESAT-6 (Abcam Inc) in PBS followed by incubation for 30 min at room temperature. Subsequently, the bacteria were washed three times with 600 µL PBS. After the last washing step, the pellet was resuspended in a 1:167 dilution of FITC-conjugated polyclonal anti-mouse IgG secondary antibody (Sigma-Aldrich) in PBS, followed by a 30-minute incubation at room temperature, protected from light. The cells were washed four times with PBS, diluted to a suitable cell density and analyzed using a MACSQuant analyzer (Miltenyi Biotec GmbH, Bergisch Gladbach, Germany). The data was processed using the FlowJo software (BD bioscience, Franklin Lakes, NJ).

### Electronic supplementary material

Below is the link to the electronic supplementary material.

Additional file 1: Contains Table S1 listing all primers used in the present study

Additional file 2: Contains Figure S1 illustrating the BUSCO results

## Data Availability

No datasets were generated or analysed during the current study.
