# Supplementary material for: Antigen surface display in two novel whole genome sequenced food grade strains, Lactiplantibacillus pentosus KW1 and KW2
Source: Microb Cell Fact. 2024 Jan 11;23:19. doi: 10.1186/s12934-024-02296-2 (PMC10782763; doi:10.1186/s12934-024-02296-2)
Supplement: Supplementary file 1 — Additional file 1: Contains Table S1 listing all primers used in the present study [file 12934_2024_2296_MOESM1_ESM.docx]

**Additional file 1**

**Supplementary Table 1**. Primers used in the present study.

| **Primer name** | **Sequence (5’🡪3’)*^a^*** | **Description** |
| --- | --- | --- |
| 11F | TAACACATGCAAGTCGAACG | Forward primer for amplification of the variable region of the 16s rRNA. |
| 5R | GGTTACCTTGTTACGACTT | Reverse primer for amplification of the variable region of the 16s rRNA. |
| KW_  Lipo-02310-F | GGAGTATGATT*CATATG*CGTTTTAAATCATTATTCATCCTACC | InFusion forward primer for amplification of the Lipoprotein anchor of *NGP02_02310* of KW1. |
| KW_  Lipo-02310-R | GACGACTAAA*GTCGAC*CGCCGCAATCGTGCCCTTAGCGGCTTGACTCGT | InFusion reverse primer for amplification of the Lipoprotein anchor of *NGP02_02310* of KW1. |
| KW1_  NTTM-02010-F | GGAGTATGATT*CATATG*CGAGTTCAACGTAGAAGGC | InFusion forward primer for amplification of the N-terminal transmembrane anchor of *NGP02_02010* of KW1. |
| KW1_  NTTM-02010-R | GACGACTAAA*GTCGAC*CGCCGCAATCGTGCCCAGCGCGTAGACTGGAACGT | InFusion reverse primer for amplification of the N-terminal transmembrane anchor of *NGP02_02010* of KW1. |
| KW2_  NTTM-12625_F | GGAGTATGATT*CATATG*CAAAATAATGGTTTTTGGGC | InFusion forward primer for amplification of the N-terminal transmembrane anchor of *LPKW2_12625* of KW2. |
| KW2_  NTTM-12625_R | GACGACTAAA*GTCGAC*CGCCGCAATCGTGCCCACCGCATTTTGCAAGTT | InFusion reverse primer for amplification of the N-terminal transmembrane anchor of *LPKW2_12625* of KW2. |
| KW1_  LysM-07240-F | GGAGTATGATTCATATGAAAAAATTATTAACCACAATCTTAACAACT | InFusion forward primer for amplification of the LysM-motif of *NGP02_07240* of KW1. |
| KW1_  LysM-07240-R | GACGACTAAA*GTCGAC*ATATAATGCCCAGGCTTGCA | InFusion reverse primer for amplification of the LysM-motif of *NGP02_07240* of KW1. |
| KW2_  LysM-06475_F | GGAGTATGATT*CATATG*AAAATCAAACACCTCTTATTATCC | InFusion forward primer for amplification of the LysM-motif of *LPKW2_06475* of KW2. |
| KW2_  LysM-06475_R | GACGACTAAA*GTCGAC*GTACCAACCGTTAGCTTGCCA | InFusion reverse primer for amplification of the LysM-motif of *LPKW2_06475* of KW2. |
| Sp-KW1_  06930_F | GGAGTATGATT*CATATG*ACAAAGGCACTAAAAGTTGCA | InFusion forward primer for amplification of the N-terminal signal peptide of *NGP02_06930* of KW1. |
| Sp-KW1_  06930_R | TTGGATAAAA*GTCGAC*GATGGCCGCACTGGCAGCG | InFusion reverse primer for amplification of the N-terminal signal peptide of *NGP02_06930* of KW1. |
| LPxTG-KW1_  06930-F | TCAGTTCCACACGCGTGCTAACGCCGATATTGAA | InFusion forward primer for amplification of the LPxTG anchor of *NGP02_06930* of KW1. |
| LPxTG-KW1_  06930-R | CTGTAATTTG*AAGCTT*TTAATCAGTCGTGTGACGT | InFusion reverse primer for amplification of the LPxTG anchor of *NGP02_06930* of KW1. |

*^a^*Restriction sites are in italics.
