# Supplementary material for: Antigen surface display in two novel whole genome sequenced food grade strains, Lactiplantibacillus pentosus KW1 and KW2
Source: Microb Cell Fact. 2024 Jan 11;23:19. doi: 10.1186/s12934-024-02296-2 (PMC10782763; doi:10.1186/s12934-024-02296-2)
Supplement: Supplementary file 2 — Additional file 2: Contains Figure S1 illustrating the BUSCO results [file 12934_2024_2296_MOESM2_ESM.docx]

**Additional file 2**


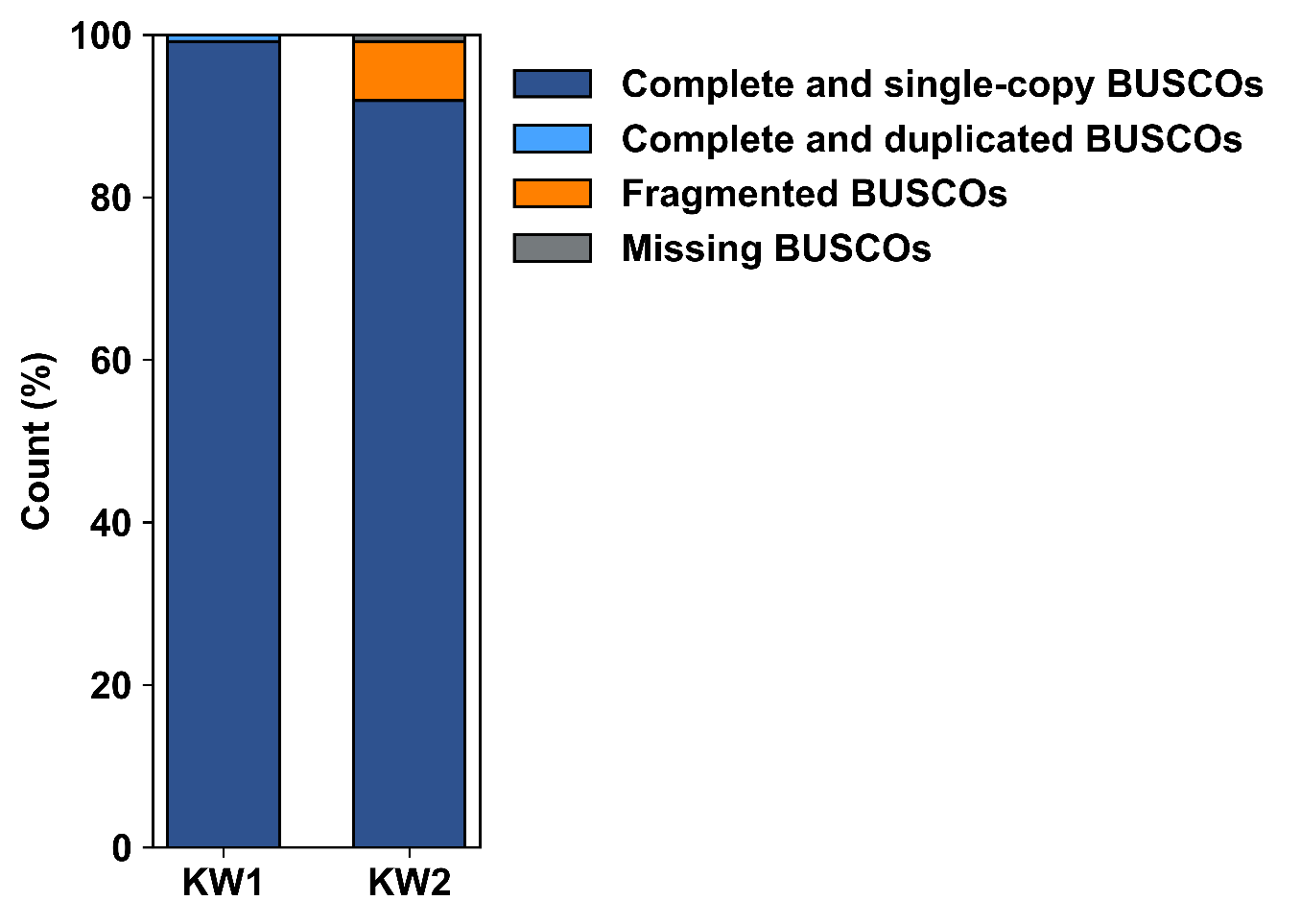


**Supplementary figure 1.** Genome assembly quality of *L. pentosus* KW1 and KW2 were assessed using the BUSCO tool, which uses Benchmarked Universal Single-Copy Orthologues to estimate completeness. We searched for orthologues against the “bacteria_odb10” dataset, which includes 124 BUSCO markers. For KW1 and KW2 respectively, the percentages of BUSCOs in each category were as follows; complete and single-copy BUSCOs: 99.2% and 91.9%, Complete and duplicated BUSCOs: 0.8% and 0%, fragmented BUSCOs: 0% and 7.3%, Missing BUSCOs: 0% and 0.8%.
